# Family-centered nursing care model for neonatal intensive care*

**DOI:** 10.1590/1980-220X-REEUSP-2025-0356en

**Published:** 2026-03-23

**Authors:** Jaquiele Jaciára Kegler, Eliane Tatsch Neves, Maria Ribeiro Lacerda

**Affiliations:** 1 Universidade Federal de Santa Maria, Santa Maria, RS, Brazil.; 2 Universidade Federal do Paraná, Curitiba, PR, Brazil.

**Keywords:** Nursing, Family, Intensive Care, Neonatal, Models, Nursing, Qualitative Research

## Abstract

**Objective:**

To build a family-centered nursing care model for neonatal intensive care.

**Method:**

This qualitative research, with a participatory approach, developed all the components of a care model based on data produced in remote discussion groups with members of a research group in the field of child health, the theoretical framework of family-centered care, and the practical experience of the study's researchers.

**Results:**

A care model was developed consisting of six concepts – newborn, family, neonatal intensive care unit, health-illness, Nursing and family-centered care –, six assumptions and six phases – getting acquainted, getting to know each other, connecting, recognizing the family's needs and strengths, developing care in partnership with the family, and rethinking the process.

**Conclusion:**

This model could guide the practice of nurses in caring for families in neonatal units and guide the training of future professionals.

## INTRODUCTION

Family-Centered Care (FCC) refers to an approach to the planning, delivery, and evaluation of health care that is based on mutually beneficial partnerships among patients, families, and professionals^(1)^. In pediatrics, this implies that the responsibility for care is shared by healthcare professionals and the family^(2)^.

This approach to care has four central pillars that should be applied at any level of assistance and in neonatal, pediatric, and adult contexts: dignity and respect, information sharing, participation, and collaboration. To achieve this, professionals must listen to and respect the choices and perspectives of patients and their families (dignity and respect); provide useful, complete, and unbiased information (information sharing); support and encourage family members to participate in care and decision-making at the level of their choice (participation); and contribute to the development, implementation, and evaluation of policies and programs with patients and families (collaboration)^(1)^.

The benefits of FCC, both for newborns (NB) and their parents, are well documented in the literature. For babies, these benefits include: better feeding outcomes and neurodevelopmental scores, greater weight gain, reduced risk of retinopathy of prematurity, shorter hospital stays, and lower hospital readmission rates; and for parents: greater involvement, less anxiety, depression and stress, greater satisfaction, and a better quality of life^(3,4)^. Despite this, FCC is still poorly understood and implemented in Neonatal Intensive Care Units (NICUs) in our country^(5,6,7)^. Therefore, it was decided that a care model that could guide and theoretically support the practice of nurses in caring for families in the NICU would be developed.

A nursing care model is a theoretical framework, represented in a diagram, composed of the four basic concepts of Nursing (environment/society, Nursing, human being, and health/illness) and others that may be necessary for its understanding; by assumptions based on a theoretical-philosophical framework; and by a care methodology, whose function is to guide nursing care through the systematization of actions^(8)^.

Care models are tools that can be used to guide nursing practice and, therefore, demystify the idea that theory and practice are not linked^(8)^. In addition to contributing to the development of differentiated and specific care, they also promote the knowledge and development of the Nursing course itself^(9)^.

Therefore, considering the gaps in professional training, with the superficial approach to FCC in undergraduate nursing courses—a fact observed in my academic and professional experience—the insufficient continuing education, and the difficulties faced by nursing professionals in developing family care in neonatal units^(5,6,7)^ and the importance of the FCC for the family and the NB, the development of a care model is justified. This model can not only guide the practice of nurses regarding family care in the NICU, but also guide the training of future professionals. Furthermore, the basic concepts for applying FCC in practice are general, meaning they do not encompass all the specificities of a neonatal unit, and they were also developed based on the experience of North American researchers, which may hinder their application in the Brazilian context, considering the country’s cultural and economic diversity.

Another justification is the lack of a model of this nature in the Brazilian context, confirmed by an integrative review study conducted in the LILACS, MEDLINE, PubMed, and WoS databases in November 2022, with the aim of identifying what has been produced about FCC in neonatal units^(10)^. In this regard, the objective of this study was to build a family-centered nursing care model for neonatal intensive care.

### Theoretical Framework

The publication entitled “*Family-centered care for children with special health care needs*”^(11)^ and the concepts of the *Institute for Patient- and Family-centered Care* ^(1)^ ground the theoretical framework of this study. In the publication “*Family-centered care for children with special health care needs”*, the authors present eight key elements for implementing the FCC for families of children with special needs, namely: Recognize that family is the constant in a child’s life, while health services and professionals are transient; Facilitate collaboration between parents and professionals at all levels of health care; Continuously share impartial and complete information with parents about the appropriate and supportive care of their children; Implement appropriate policies and programs that are comprehensive and provide emotional and financial support to meet the needs of families; Recognize the strengths and individuality of the family and respect different coping methods; Understand and incorporate the developmental needs of infants, children, and adolescents and their families into health systems; Encourage and facilitate parent-to-parent support; and Ensure that the Health services design be flexible, accessible, and responsive to the family’s needs^(11)^.

These elements formed the basis for the construction of the concepts of Patient-and Family-Centered Care (PCFC) – dignity and respect, information sharing, participation, collaboration – presented by *Institute for Patient- and Family-centered Care*. These four concepts are described below:

– Dignity and respect: Healthcare professionals listen to and honor the perspectives and choices of the family and patient. The knowledge, values, beliefs, and cultural backgrounds of the family and patient are incorporated into the planning and delivery of care^(1)^;
– Information sharing: Healthcare professionals communicate and share complete and unbiased information with patients and families in an affirmative and helpful manner. Patients and families receive complete, up-to-date, and accurate information to effectively participate in care and decision-making^(1)^;
– Participation: Patients and families are encouraged and supported to participate in care and decision-making at the level they choose^(1)^;
– Collaboration: Patients, families, healthcare professionals, and leaders collaborate in the development, implementation, and evaluation of policies and programs; in research; in the design of healthcare services; and in professional education, as well as in the promotion of care itself^(1)^.

Two other important definitions are those of family and of the PCFC itself. In the definition presented by *Institute for Patient- and Family-centered Care* (2024)^(1)^ patients and their families themselves define what their family is. PCFC refers to an approach to the planning, delivery, and evaluation of health care that is based on mutually beneficial partnerships among patients, families, and professionals^(1)^.

## METHOD

### Design of Study

This is a qualitative research study with a participatory approach, in which all the components of a care model were developed, namely: the concepts, the assumptions, the care methodology, and a diagram.

### Population and Selection Criteria

For participant selection, the inclusion criteria were: being a nurse or undergraduate student in Nursing and having at least two years of participation in the Neonatal, Child, Adolescent and Family Health research group (CRIANDO). Students who had not completed the sixth semester of the Nursing course were excluded. This criterion was adopted considering that the students are taking courses related to child and family care this semester.

To identify those who met the inclusion criteria, a table was created with the names of all members of the research group. Next, these individuals were contacted via messaging application (WhatsApp), and each was asked about their length of participation in the group and, if they were undergraduates, which semester they were currently in. Subsequently, all members who met the inclusion criteria were invited by email.

### Data Collection

Data were collected from remote focus groups conducted with members of the CRIANDO research group. The focus group is a data collection technique through which the researcher seeks the collective construction of ideas^(12)^.

Upon acceptance, an email was sent three days prior to the scheduled date for the discussion group, containing information such as the purpose of the group, date, time and access link, the free informed consent form, as well as the activity that should be carried out beforehand – each participant should choose three keywords for each concept (family, NB, nursing, NICU, health-disease and FCC), which in their opinion represented it. The chosen keywords were presented by each participant on the day scheduled for each group.

Two separate groups were organized, both with seven members, who met in the evening of December 2020 using the Google Meet application. The discussion in group I lasted one hour and eight minutes, and the discussion in group II lasted one hour and forty-three minutes. Both groups included participants at all academic levels – doctoral, master’s, and undergraduate.

 In the groups, after obtaining consent, the participants were introduced, and the group’s objective and how it would be organized were explained. Subsequently, each participant presented the three words they had chosen for each concept, explaining their choice. At the end of the discussion of each concept, the researcher compiled a summary of the participants’ keywords, which were then compiled into a Power point file by the research assistant. To maintain organization, the presentation, discussion, and synthesis were carried out by concept, following the order: NB, NICU, and so on. The groups were coordinated by the researcher, author of the thesis project, and included the participation of a previously trained research assistant, who was an undergraduate student in Nursing and also a scholarship recipient of the project.

### Data Analysis and Treatment

The audio recordings from the discussion groups were transcribed into a Word document and analyzed, using inductive thematic analysis as a reference^(13)^, following these steps:

– Familiarization with the data: exhaustive readings and rereadings of the transcribed material were carried out, and the keywords for each concept in the text were highlighted. For this, six different colors were used;
– Generation of initial codes: all keywords highlighted in the text were grouped into a table, read exhaustively, and categorized by color according to thematic affinity;
– Theme generation: keywords highlighted in the same color were grouped into themes;
– Reviewing the themes: the themes were reviewed to confirm whether the keywords grouped under each theme adequately represented it;
– Concept generation: the themes of each concept were grouped and, with the aid of connectives in the Portuguese language, as well as based on the theoretical framework of the thesis, the concepts were constructed. The analytical process that underpinned the development of each of the concepts is detailed in an article published in the Brazilian Journal of Nursing^(14)^.

Subsequently, two more focus groups were held to validate the concepts developed and/or to suggest changes. The group meetings were held in June 2021 via Google Meet, with 11 of the 14 members from the previous groups participating. At this time, 3 of them were unable to participate for personal reasons. Group I (five members) had a duration of one hour and fifteen minutes, and Group II (six members) had a duration of one hour and eleven minutes. In these groups, participants presented their suggestions to the others and analyzed which aspects of the concepts required adjustments. It should be noted that the concepts were previously sent via email for review. In all cases, modifications were suggested, which were analyzed and discussed by the research team, consisting of the doctoral student, advisor, and co-advisor, who again reformulated the concepts.

The assumptions and methodology of care were developed based on the theoretical framework of the thesis^(1,11)^ and the researchers’ practical experience. Throughout the research team’s meetings, the components of the care model were reformulated to reflect the theoretical framework and to be clear, consistent with each other, and with practice.

After its development, the concepts, assumptions, and methodology of care were sent for grammatical review, so that textual cohesion and coherence could be analyzed. These were then restructured by the research team based on the reviewer’s suggestions.

**Figure 1:**
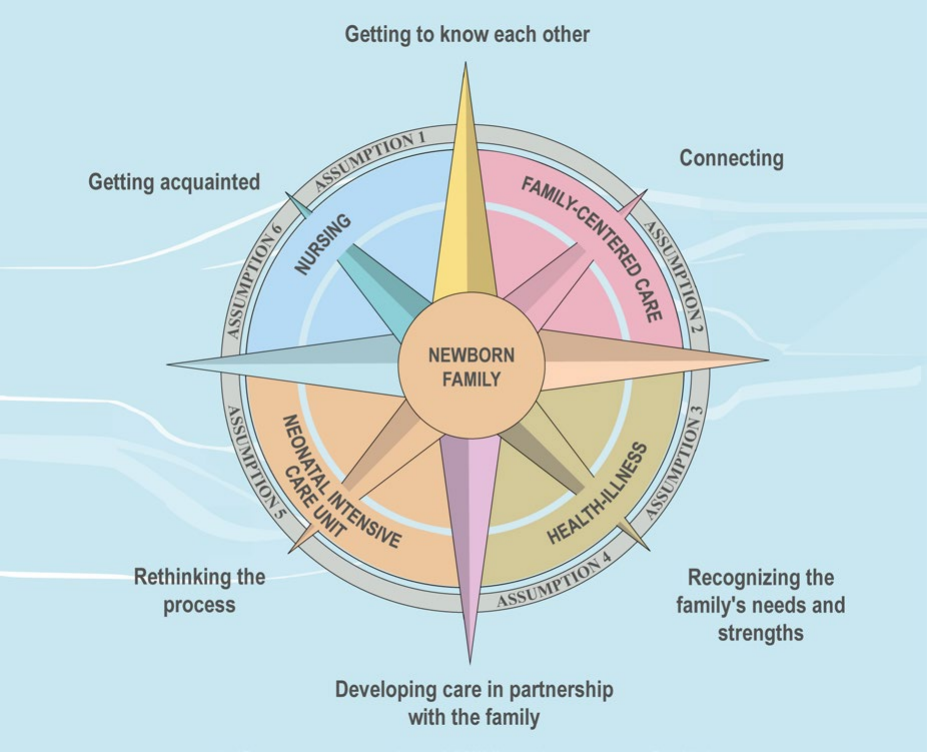
Graphic representation of the family-centered nursing care model for neonatal intensive care (AMCORE).

A diagram, as an element of a nursing care model, was also developed. Thus, during the process of developing the concepts, the researcher had drawn on an A4 sheet a preliminary diagram of the first insights of how the concepts should be related. Later, on the day of the focus groups, when the concepts were validated, the participants also presented and explained the diagram they had drawn to represent the concepts. It should be noted that in the same email in which the Word file was sent with the concepts presented, participants were asked, as a task to be completed prior to the group session, to draw on an A4 sheet how they would represent the concepts in a diagram and to send it via the messaging application (WhatsApp). These diagrams served as inspiration, along with the ideas discussed by the research team, for the creation of the illustration (Figure 1), representing the care model, made by a professional graphic designer hired by the doctoral researcher.

After the care model was developed, some revisions were made to ensure it was easy to understand, simple, and attractive to nurses. For this purpose, colors and illustrations were used to represent the phases of the methodology, also created by a graphic designer, as well as some deletions and additions of definitions of the components of a care model. The goal was to create material of interest to nurses.

### Ethical Aspects

The study was developed following Resolution No. 466/2012 and approved by the Research Ethics Committee, under opinion number 3.485.858.

## RESULTS

Figure 1 shows a graphical representation of the family-centered nursing care model for neonatal intensive care. The model is formed by six concepts – NB, family, NICU, health-illness, nursing, and FCC – and six assumptions: 1) Nursing should make the NICU accessible, flexible, and responsive to the family’s needs; 2) Nursing should promote the central role of the family in newborn care, respecting the opinions, choices, and uniqueness of family members; 3) Building a bond between nurse and family is essential for the development of FCC; 4) The nurse needs to know the family to identify their care needs, coping strategies, and strengths; 5) For effective communication between nurse and family, the professional must be open, available, and without prejudice during dialogue; 6) Partnership between family and healthcare professionals should be encouraged for the development of care in a participatory manner – and six phases – getting acquainted, getting to know each other, connecting, recognizing the family’s needs and strengths, developing care in partnership with the family, and rethinking the process.

To simplify the model’s name, the acronym AMCORE was chosen to represent it. It is noteworthy that this acronym was created from the first syllables of the names (in Portuguese) of the phases of the care methodology: AM for getting acquainted (*ambientando-se*), CO for getting to know each other and connecting, and RE for recognizing the family’s needs and strengths. Furthermore, the acronym AMCORE refers to what is expected of this model: “anchoring,” that is, establishing a bond and supporting the family in the NICU.

The diagram was inspired by a compass rose, considering that the model serves as a guide, a direction for family care in the NICU. The NB and the family are at the center, as they are the recipients of the care supported by the model, and the other concepts revolve around them. The phases of the methodology are at the tips of the rose, as they represent the directions to be followed for family care. Chart 1 shows the definitions of each of the concepts represented in the diagram.

**Chart 1:** Definitions of the concepts that comprise the family-centered nursing care model for neonatal intensive care (AMCORE) – Santa Maria, RS, Brasil, 2026.

**NEWBORN** It is a being welcomed into the family, which alters its dynamics, generating expectations and concerns. It is a fragile being that needs family care and, sometimes, care from healthcare professionals.
**FAMILY-CENTERED CARE** It is broad and complex, requiring connection, respect for the family perspective, effective communication, and active participation from both the family and the multidisciplinary team.
**NURSING** It utilizes the science of care, acting through assistance, management, health education, and research. In the neonatal intensive care unit, the goal is to welcome the newborn and its family, with the aim of promoting, maintaining, or restoring health, and to become a point of reference for care within the multidisciplinary team.
**NEONATAL INTENSIVE CARE UNIT** It is a therapeutic environment, equipped with technology and a multidisciplinary team, which provides care to the newborn and their family. This environment evokes ambiguous feelings such as insecurity/security and fear/hope.
**HEALTH-ILLNESS** It is a subjective experience for the family, as it depends on their history and living conditions. It is experienced by the newborn and their family, and can generate feelings and expectations that need to be considered when providing care.

In their assumptions, the researchers focused on highlighting the importance of the nurse in the implementation of each of them. It should be noted that these were constructed deductively, based on the eight elements discussed in the publication “*Family-centered care for children with special health care needs*”^(11)^ as well as the researcher’s experience as a nurse in a pediatric intensive care unit.

**Figure 2:**
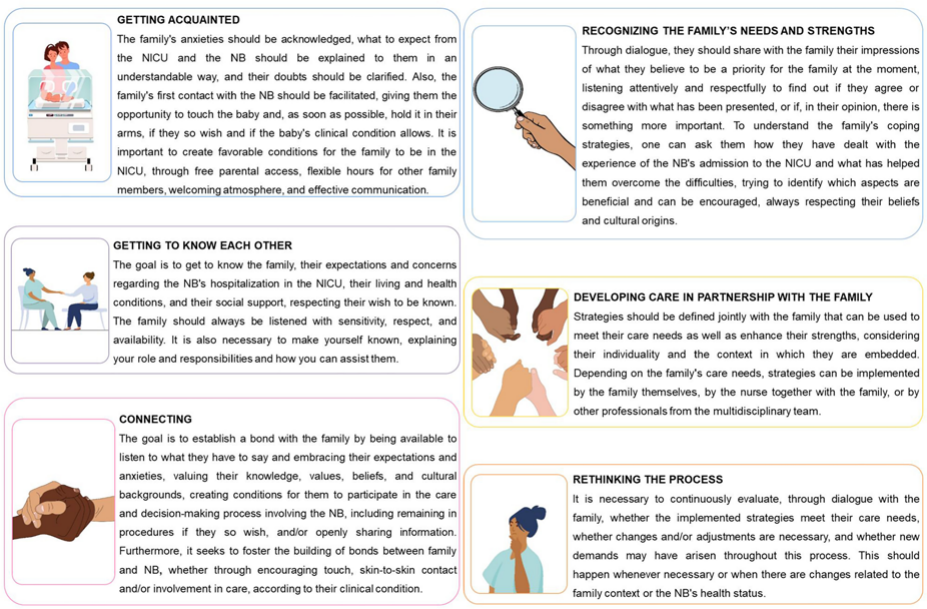
Methodology of care for the family-centered nursing care model for neonatal intensive care (AMCORE).

The phases of the care model methodology were developed with the aim of systematizing how family care should be provided in the NICU. Other care models were used as a theoretical basis for this stage^(8,9,15,16)^, which shed light on which aspects would be important to address in each phase and, above all, the theoretical framework of the study^(1,11)^, supplemented by the researcher’s practical experience. In all phases, the timing of their execution and the actions that can be developed to address them are indicated. The goal is not for them to be watertight, but rather flexible to the NICU in which they are being developed. Therefore, the methodology presented seeks to offer pathways for family care in the NICU. Moreover, in outlining the phases, the authors of the study were concerned with reinforcing the importance of the family being involved in the NICU environment and in the care of the NB, given that they still need to build a bond with the newborn, which occurs after birth, and thus develop their parental role, necessary for the care of the NB. Figure 2 presents the care methodology of the model.

The *getting acquainted* was listed as the first phase because we believe that the family needs this initial period to get used to the NICU environment and to the NB, in addition to the necessary acclimatization by the nurse, since it is understood that each family is unique and must be cared for in a singular way.

Starting from this phase, we have the *getting to know each other,* in which the nurse seeks to get to know the family and to make themselves known, to begin building the trust necessary for caring for the family. Next, in the phase *connecting us,* the nurse acts to foster the building of a bond between the nurse and the family, and between the family and the NB. Based on the information obtained in the phases *getting acquainted* and *getting to know each other,* the nurse shares the identified needs with the family through open dialogue; this is the phase *recognizing the family’s needs and strengths*.

In the next phase, care is developed in partnership with the family, defining, together with them, strategies that can be developed to meet their care needs and enhance their strengths. In the phase *rethinking the process,* the nurse assesses whether the implemented strategies met the family’s care needs, whether changes and/or adjustments are necessary, and whether new demands may have arisen during this process.

It is important to note that, although the phases are described in a specific order, they are not linear because, at times, the nurse will need to return to a previous phase before advancing to the next, or may even be in the fifth phase and need to return to the third. However, for a FCC to occur, it is essential that all phases are observed, implemented, reconsidered, and reflected upon.

## DISCUSSION

A care model should consist of the concepts of the Nursing metaparadigm and others that may be necessary. The metaparadigm is the most abstract component of the nursing knowledge framework. It is defined as the global concepts that identify the central phenomenon of interest to the discipline, the propositions that describe the concepts, and those that establish the relationships between them. The concepts that comprise the metaparadigm of Nursing are human being, environment, health, and Nursing^(17)^.

In this way, the concepts of the metaparadigm were developed, in which the concept of NICU refers to the environment, and the concepts of newborn and family refer to the human being. Furthermore, the concept of FCC was developed considering its relevance to the constructed model.

The model’s assumptions are consistent with the attributes identified in the study, which analyzed the concept of FCC in NICUs. Attributes are characteristics that make concepts unique in relation to others. The attributes of the FCC concept in NICUs identified are: family care – involves identifying and meeting the family’s care needs; equal family participation – the family participates in planning and providing care and in decision-making; collaboration – professionals cooperate with families in the development and implementation of care plans; maintaining respect and dignity for the family – professionals recognize the differences between families, including them in the development of care plans; and information – professionals share complete information with families according to their specific needs^(18)^.

The phases of the care methodology were developed to serve as a guide for family care in the NICU, comprising a set of interconnected phases – complementary, simultaneous or not – that seek to guide nursing practice. In the phase *getting acquainted*, the goal is to familiarize the family with the NICU and the NB environment. When parents enter this unit, they encounter an unfamiliar and frightening environment, surrounded by equipment and the image of a NB different from the one idealized during pregnancy, with many devices and equipment, which makes initial contact with the baby difficult. This implies that the team’s professionals support the family, helping them overcome difficulties in consolidating the bond^(19)^. At this stage, the nurse should embrace the family, explain about the unit and the NB in an understandable way, clarify their doubts, and show that they are available to listen to them.

Another study confirms that, upon admission of the NB to the NICU, the family is also embraced, at which time they are informed about the NB’s health status, given instructions on the unit’s routines, and have their questions answered^(20)^. Parents need to know about NICU routines, medical devices, and their child’s health condition, which can alleviate their fear of the unknown, their anxiety about the uncertainty of the situation, and their feelings of exclusion and helplessness^(21)^.

Furthermore, the nurse should facilitate the family’s first contact with the NB, giving the family member the opportunity to touch the baby and, as soon as possible, hold it in their arms. The initial contact between mother and newborn is important for the child’s physical, psychological, and intellectual development. The admission of a newborn to the NICU can compromise the fragile bond created between them. Physical contact, whether through touch, holding, or kangaroo care, has been cited as necessary for parents to develop bonding and their parental role^(22,23)^.

Conditions to encourage family presence in the NICU should be implemented, such as: free access for parents, flexible hours for other family members, a welcoming environment, and effective communication. A study conducted in a NICU in New Zealand indicated that the lack of an unrestricted visitation policy for parents and the lack of childcare options for the other children hinders their presence in the unit^(24)^.

To plan effective care, it is essential to know the family. Thus, in the phase *getting to know each other*, the goal is to get to know the family, their expectations and concerns regarding the NB’s hospitalization in the NICU, their living and health conditions, and their social support. It is important to listen to it with sensitivity, respect, and openness, because this phase involves much more than just compiling a family history; it is about listening to what each family member has to share at that moment. Other models^(15,25)^ have, among their phases, the one in which the nurse seeks to get to know the person being cared for, confirming the importance of this phase for a model.

Furthermore, at this stage, the nurse introduces themselves, explaining their role and responsibilities to the family, and how they can assist them. A study found that parents did not know who the nurses were or what their responsibilities were^(26)^. Therefore, it is necessary for nurses to introduce themselves to the family, so that the family knows what these professionals’ responsibilities are and their role within the multidisciplinary team, thus contributing to the visibility of Nursing within the unit.

In the phase *connecting*, the goal is to establish a bond with the family, as this is known to be fundamental for the development of the FCC^(7)^. This bond can be built through the nurse’s willingness to listen to the family, acknowledging their expectations and anxieties, valuing their knowledge, values, beliefs, and cultural backgrounds, creating conditions for them to participate in the care and decision-making process involving the NB and/or openly sharing information. When parents feel supported and trust healthcare professionals, it reduces the stress caused by NICU admission and increases their confidence and ability to care for their child^(27)^.

In the phase *recognizing the family’s needs and strengths*, the nurse establishes the care priorities for family members, based on information obtained in the phases *acquainted* and *getting to know each other*. Through dialogue, they share with the family their impressions of what they believe to be a priority for them at the moment, listening attentively and respectfully to find out if they agree or disagree with what has been presented, or if, in their opinion, there is something more important. When seeking FCC, it is important to keep in mind that the family’s perspectives and choices should always be heard and respected^(1)^. It is equally important at this phase to recognize the family’s coping strategies and strengths, which can be encouraged and strengthened to help them overcome the challenges arising from the NB’s hospitalization in the NICU.

In the phase *developing care in partnership with the family*, the goal is to define, together with the family, the strategies that will be used to meet their care needs as well as to enhance their strengths. Depending on the family’s care needs, strategies can be implemented by the family themselves, by the nurse together with the family, or by other members of the multidisciplinary team.

The studies focus on the partnership that must be developed with parents so that they can provide care for the NB, thus contributing to the development of their skills, reducing the fear and insecurity of caring for their child at home^(2,28,29)^. However, the partnership sought at this stage goes beyond the care of the newborn; it aims to meet the needs of the family, considering its individuality and the context in which it is embedded. Only in this way can we fulfill the purpose of the FCC, which is to care for the family by involving its members in the planning and execution of care^(1)^.

The phase *rethinking the process* takes place when the nurse, together with the family, assesses whether the strategies implemented in the previous phase met their care needs, whether changes and/or adjustments are necessary, or whether new demands may have arisen. It is known that the admission of a NB to the NICU is marked by uncertainties and that their condition can change at any moment, and the nurse must be sensitive to this to assist the family in changes^(19)^.

The study’s limitations relate to the fact that data collection was carried out remotely and in a study setting in southern Brazil. Among the contributions to the advancement of scientific knowledge in the field of health and nursing, it is noteworthy that the care model constructed in this study is an innovative tool for the dissemination of FCC in NICUs, since its care methodology offers elements that can guide nursing professionals in the care of families in the neonatal intensive care environment, in a simple way that is adaptable to the reality of each health service. Furthermore, the study provides necessary visibility to the fact that, to develop quality, humanized, and participatory care for the families of newborns hospitalized in the NICU, theory and practice must go hand in hand.

## CONCLUSION

AMCORE consists of the concepts of family, NB, FCC, nursing, NICU, and health-disease, based on assumptions rooted in a theoretical-practical framework and a care methodology composed of six phases, which seeks to systematize care for families in NICUs. It is represented by a diagram and aims not only to guide the practice of nurses in neonatal units regarding family care, but also the training of future professionals, aiming for the FCC principles to be increasingly disseminated and implemented in practice.

## Data Availability

The entire dataset supporting the results of this study has been made available in a Data Repository: http://repositorio.ufsm.br/handle/1/29428.
