# Supplementary material for: Family-centered nursing care model for neonatal intensive care
Source: Rev Esc Enferm USP. 2026 Mar 23;60:e20250356. doi: 10.1590/1980-220X-REEUSP-2025-0356en (PMC13038217; doi:10.1590/1980-220X-REEUSP-2025-0356en)
Supplement: Translation

# Translation: Modelo de cuidado de enfermagem centrado na família para terapia intensiva neonatal*

**DOI:** 10.1590/1980-220X-REEUSP-2025-0356pt

**Authors:** Jaquiele Jaciára Kegler, Eliane Tatsch Neves, Maria Ribeiro Lacerda

**Affiliations:** 1 Universidade Federal de Santa Maria, Santa Maria, RS, Brasil.; 2 Universidade Federal do Paraná, Curitiba, PR, Brasil.

**Keywords:** Enfermagem, Família, Terapia Intensiva Neonatal, Modelos de Enfermagem, Pesquisa Qualitativa

## Abstract

**Objetivo:**

Construir um modelo de cuidado de enfermagem centrado na família para terapia intensiva neonatal.

**Método:**

Pesquisa qualitativa, com abordagem participatória, em que foram desenvolvidos todos os componentes de um modelo de cuidado, a partir dos dados produzidos nos grupos de discussão remotos com integrantes de um grupo de pesquisa da área da saúde da criança, do referencial teórico de cuidado centrado na família e da experiência prática das pesquisadoras do estudo.

**Resultados:**

Desenvolveu-se um modelo de cuidado constituído por seis conceitos – recém-nascido, família, unidade de terapia intensiva neonatal, saúde-doença, Enfermagem e cuidado centrado na família –, seis pressupostos e seis fases – ambientando-se, conhecendo-nos, conectando-nos, reconhecendo as necessidades e pontos fortes da família, desenvolvendo o cuidado em parceria com a família e repensando o processo.

**Conclusão:**

O modelo poderá guiar a prática de enfermeiros no cuidado de famílias em unidades neonatais e orientar a formação de futuros profissionais.

## INTRODUÇÃO

O Cuidado Centrado na Família (CCF) refere-se a uma abordagem para o planejamento, prestação e avaliação dos cuidados de saúde, que se baseia em parcerias mutuamente benéficas entre pacientes, famílias e profissionais^(1)^, Em pediatria, isso implica que a responsabilidade do cuidado seja compartilhada pelos profissionais de saúde e família^(2)^.

Esse cuidado apresenta quatro pilares centrais que devem ser aplicados em qualquer nível de assistência e nos contextos neonatal, pediátrico e adulto: a dignidade e o respeito, o compartilhamento de informações, a participação e a colaboração. Para isso, os profissionais devem ouvir e respeitar as escolhas e perspectivas dos pacientes e de suas famílias (dignidade e respeito); fornecer informações úteis, completas e imparciais (compartilhamento de informações), apoiar e estimular os familiares a participarem dos cuidados e das tomadas de decisões no nível de sua escolha (participação); e contribuir com os pacientes e familiares no desenvolvimento, implantação e avaliação de políticas e programas (colaboração) ^(1)^.

Os benefícios do CCF, tanto para os Recém-Nascidos (RN) como para os seus pais, estão bem documentados na literatura. Para os bebês, esses benefícios são: melhores resultados na alimentação e nos escores de neurodesenvolvimento, maior ganho de peso, diminuição do risco de retinopatia da prematuridade, menor tempo de internação e menores taxas de readmissão hospitalar; e para os pais: maior participação, menos ansiedade, depressão e estresse, maior satisfação e melhor qualidade de vida^(3,4)^. Apesar disso, o CCF ainda é pouco compreendido e implementado nas Unidades de Terapia Intensiva Neonatais (UTIN) do nosso país^(5,6,7)^. Por isso, decidiu-se pelo desenvolvimento de um modelo de cuidado que pudesse direcionar e fundamentar teoricamente a prática de enfermeiros no cuidado das famílias em UTIN.

Um modelo de cuidado de enfermagem é um conjunto teórico, representado em um diagrama, composto pelos quatro conceitos básicos da Enfermagem (meio ambiente/sociedade, Enfermagem, ser humano e saúde/doença) e outros que se fizerem necessários para o seu entendimento; por pressupostos que têm por base um referencial teórico-filosófico; e por uma metodologia do cuidado, cuja função é nortear o cuidado de enfermagem por meio da sistematização das ações^(8)^.

Os modelos de cuidado são ferramentas que podem ser utilizadas para guiar a prática de enfermeiros e, por conseguinte, desmitificam a ideia de que teoria e prática não se articulam^(8)^. Além de contribuírem para o desenvolvimento de um cuidado diferenciado e específico, impulsionam também o próprio conhecimento e desenvolvimento da disciplina de Enfermagem^(9)^.

Dessa forma, considerando-se as lacunas na formação profissional, ao abordarem o CCF de forma superficial nas disciplinas da graduação em enfermagem, fato constatado na minha vivência acadêmica e profissional, a educação permanente insuficiente, as dificuldades dos profissionais de enfermagem em desenvolver o cuidado da família em unidades neonatais^(5,6,7)^ e a importância do CCF para a família e o RN, justifica-se a construção de um modelo de cuidado que possa não só guiar a prática de enfermeiros no que se refere ao cuidado da família em UTIN, mas também orientar a formação de futuros profissionais. Além disso, os conceitos básicos para a aplicação do CCF na prática são gerais, ou seja, não abarcam todas as especificidades de uma unidade neonatal e, ainda, foram desenvolvidos a partir da experiência de pesquisadores norte-americanos, o que pode dificultar a sua aplicação no cenário brasileiro, considerando a diversidade cultural e econômica do país.

Outra justificativa é a inexistência de um modelo desta natureza no contexto brasileiro, confirmada a partir de um estudo de revisão integrativa realizado nas bases de dados LILACS, MEDLINE, PubMed e WoS, em novembro de 2022, com o intuito de identificar o que foi produzido sobre o CCF em unidades neonatais^(10)^. Nesse sentido, este estudo teve como objetivo construir um modelo de cuidado de enfermagem centrado na família para terapia intensiva neonatal.

### Referencial Teórico

A publicação intitulada “*Family-centered care for children with special health care needs*”^(11)^ e os conceitos do *Institute for Patient-and Family-centered Care* ^(1)^ embasam o referencial teórico deste estudo. Na publicação “*Family-centered care for children with special health care needs”*, as autoras apresentam oito elementos considerados chave para a implementação do CCF para famílias de crianças com algum tipo de necessidade especial, quais sejam: Reconhecer que a família é a constante na vida da criança, enquanto os serviços e profissionais de saúde são transitórios; Facilitar a colaboração entre pais e profissionais em todos os níveis de atenção à saúde; Compartilhar continuamente informações imparciais e completas com os pais sobre o cuidado de seus filhos de maneira adequada e de apoio; Implementar políticas e programas apropriados que sejam abrangentes e forneçam apoio emocional e financeiro para atendimento das necessidades das famílias; Reconhecer os pontos fortes e individualidade da família e respeitar os diferentes métodos de enfrentamento; Compreender e incorporar as necessidades de desenvolvimento de bebês, crianças e adolescentes e suas famílias nos sistemas de saúde; Incentivar e facilitar o apoio pais-para-pais; e Garantir que o *design* dos serviços de saúde seja flexível, acessível e responsivo às necessidades da família^(11)^.

Esses elementos constituíram a base para a construção dos conceitos do Cuidado Centrado no Paciente e Família (CCPF) – dignidade e respeito, compartilhamento de informações, participação, colaboração – apresentados pelo *Institute for Patient- and Family-centered Care*. A seguir, encontram-se descritos esses quatro conceitos:

– Dignidade e respeito: Os profissionais de saúde escutam e honram as perspectivas e escolhas da família e paciente. O conhecimento, valores, crenças e origens culturais da família e paciente são incorporadas no planejamento e prestação de cuidados^(1)^;
– Compartilhamento de informações: Os profissionais de saúde comunicam e dividem informações completas e imparciais com pacientes e famílias de maneira afirmativa e útil. Pacientes e famílias recebem informações completas, atualizadas e corretas para participar efetivamente do cuidado e da tomada de decisão^(1)^;
– Participação: Pacientes e famílias são encorajadas e apoiadas a participar do cuidado e da tomada de decisão no nível que escolhem^(1)^;
– Colaboração: Pacientes, famílias, profissionais de saúde e líderes colaboram no desenvolvimento de políticas e programas, implementação e avaliação; em pesquisa; no design do serviço de saúde; e na educação profissional, bem como na promoção do cuidado em si^(1)^.

Outras duas definições importantes são a de família e do próprio CCPF. Na definição apresentada pelo *Institute for Patient-and Family-centered Care* (2024)^(1)^, os próprios pacientes e familiares definem a sua família. E o CCPF é entendido como uma abordagem para o planejamento, prestação e avaliação dos cuidados de saúde, baseada em parcerias mutuamente benéficas entre pacientes, famílias e profissionais^(1)^.

## MÉTODO

### Tipo ou Desenho do Estudo

Trata-se de uma pesquisa qualitativa, com abordagem participatória, em que foram elaborados todos os componentes de um modelo de cuidado, a saber: os conceitos, os pressupostos, a metodologia do cuidado e um diagrama.

### População e Critérios de Seleção

Para a seleção dos participantes, elegeu-se como critérios de inclusão: ser enfermeiro ou graduando em Enfermagem e possuir no mínimo dois anos de participação no grupo de pesquisa Saúde do Neonato, Criança, Adolescente e Família (CRIANDO). Foram excluídos os graduandos que não tivessem concluído o sexto semestre do curso de Enfermagem. Esse critério foi adotado considerando que os estudantes cursam as disciplinas relacionadas ao cuidado da criança e da família nesse semestre.

A fim de identificar aqueles que atendiam aos critérios de inclusão foi elaborado um quadro com os nomes de todos os integrantes do grupo de pesquisa. Em seguida, entrou-se em contato com estes, via aplicativo de mensagens (WhatsApp), questionando cada um deles sobre o tempo de participação no grupo e, quando graduando, o semestre que estava cursando no momento. Posteriormente, todos os integrantes que atendiam aos critérios de inclusão foram convidados por e-mail.

### Coleta de Dados

Os dados foram coletados a partir de grupos de discussão remotos realizados com integrantes do grupo de pesquisa CRIANDO. O grupo de discussão é uma técnica de coleta de dados, por meio da qual o pesquisador busca a construção coletiva de ideias^(12)^.

Mediante o aceite, encaminhou-se um e-mail três dias antes do dia agendado para realização do grupo de discussão, contendo informações como finalidade do grupo, dia, horário e link de acesso, o termo de consentimento livre e esclarecido bem como a atividade que deveria ser realizada previamente – cada participante deveria escolher três palavras-chaves para cada conceito (família, RN, enfermagem, UTIN, saúde-doença e CCF), que na sua opinião o representasse. As palavras-chaves escolhidas foram apresentadas por cada participante no dia agendado para cada grupo.

Foram organizados dois grupos distintos, ambos com sete integrantes, que se reuniram no mês de dezembro de 2020, no turno da noite, utilizando o aplicativo Google Meet. A discussão do grupo I teve uma duração de uma hora e oito minutos e a do grupo II, uma hora e quarenta e três minutos. Em ambos os grupos participaram todos os níveis acadêmicos – doutorado, mestrado e graduação.

 Nos grupos, após obtido o consentimento, foi realizada a apresentação dos participantes e explicado o objetivo do grupo e como ele seria organizado. Posteriormente, cada participante apresentou as três palavras que escolheu para cada conceito, explicando a sua escolha. Ao final da discussão de cada conceito, foi feita uma súmula das palavras-chaves dos participantes pela pesquisadora, que foram reunidas em um arquivo *PowerPoint* pela auxiliar de pesquisa. Para manter uma organização, foi realizada a apresentação, discussão e síntese por conceito, seguindo a ordem, RN, UTIN, e assim sucessivamente. Os grupos foram coordenados pela pesquisadora, autora do projeto da tese, e contaram com a participação de uma auxiliar de pesquisa, previamente capacitada, que era graduanda em Enfermagem e também bolsista do projeto.

### Análise e Tratamento dos Dados

Os áudios oriundos dos grupos de discussão foram transcritos em documento *Word* e analisados, tendo por referência a análise temática indutiva^(13)^, seguindo os passos:

– Familiarização com os dados: foram realizadas leituras e releituras exaustivas do material transcrito e destacadas as palavras-chaves de cada conceito no texto. Para isso, foram utilizadas seis cores diferentes;
– Geração de códigos iniciais: todas as palavras-chaves destacadas no texto foram agrupadas em um quadro, lidas exaustivamente e categorizadas por cores de acordo com a afinidade temática;
– Geração dos temas: as palavras-chaves destacadas com a mesma cor foram agrupadas em temas;
– Revisando os temas: os temas foram revisados, a fim de confirmar se aquelas palavras-chaves que estavam agrupadas em cada tema representavam-no adequadamente;
– Geração dos conceitos: os temas de cada conceito foram agrupados e, com auxílio dos conectivos da língua portuguesa, bem como tendo por base o referencial teórico da tese, construíram-se os conceitos. O processo de análise que subsidiou a elaboração de cada um dos conceitos está detalhado em artigo publicado na Revista Brasileira de Enfermagem^(14)^.

Em um segundo momento, foram realizados mais dois grupos de discussão, para a validação dos conceitos construídos e/ou sugeridas alterações. Os grupos foram realizados em junho de 2021, por meio do Google Meet, em que participaram 11 do total de 14 integrantes dos grupos anteriores. Neste momento, 3 deles não puderam participar por motivos pessoais. O grupo I (cinco integrantes) teve uma duração de uma hora e quinze minutos e o grupo II (seis integrantes), de uma hora e onze minutos. Nesses grupos, os participantes apresentaram suas sugestões aos demais e analisaram quais aspectos dos conceitos necessitavam de adequações. Destaca-se que os conceitos foram enviados previamente por e-mail para análise. Em todos, foram sugeridas modificações, que foram analisadas e discutidas pela equipe de pesquisa, constituída pela pesquisadora doutoranda, orientadora e coorientadora, que novamente reformularam os conceitos.

Os pressupostos e a metodologia do cuidado foram elaborados, tendo por base o referencial teórico da tese^(1,11)^ e a experiência prática das pesquisadoras. Ao longo dos encontros da equipe de pesquisa, os componentes do modelo de cuidado foram sendo reformulados, a fim de que refletissem o referencial teórico e estivessem claros, coerentes entre si e com a prática.

Após a sua elaboração, os conceitos, pressupostos e a metodologia do cuidado foram enviados para revisão gramatical, para que fosse analisada a coesão e a coerência textual. Em seguida, estes foram reestruturados pela equipe de pesquisa a partir das sugestões da revisora.

Um diagrama, como elemento de um modelo de cuidado de enfermagem, também foi elaborado. Desta forma, durante o processo de elaboração dos conceitos, a pesquisadora havia desenhado em uma folha A4 um diagrama preliminar dos primeiros *insights* de como os conceitos deveriam estar relacionados. Posteriormente, no dia dos grupos de discussão, em que houve a validação dos conceitos, os participantes também apresentaram e explicaram o diagrama que desenharam para representar os conceitos. Destaca-se que no mesmo e-mail em que foi enviado o arquivo *Word* com os conceitos, foi solicitado, como tarefa a ser desenvolvida previamente ao dia do grupo, que os participantes desenhassem em uma folha A4 como representariam os conceitos em um diagrama, enviando-o pelo aplicativo de mensagens (WhatsApp). Estes diagramas serviram de inspiração, juntamente com as ideias discutidas pela equipe de pesquisa para a criação da ilustração (Figura 1), representando o modelo de cuidado, feita por profissional de designer gráfico, contratado pela pesquisadora doutoranda.

**Figura 1:** Representação gráfica do modelo de cuidado de enfermagem centrado na família para terapia intensiva neonatal (AMCORE).

Após elaborado o modelo de cuidado, foram realizadas algumas reformulações para que ele fosse de fácil entendimento, simples e atrativo para os enfermeiros. Para isso, foram utilizadas cores e ilustrações para representar as fases da metodologia, criadas também pelo profissional de designer gráfico, bem como feitas algumas supressões e acrescentadas as definições dos componentes de um modelo de cuidado. O objetivo era criar um material de interesse para os enfermeiros.

### Aspectos Éticos

O estudo foi desenvolvido seguindo a Resolução nº 466/2012 e aprovado por Comitê de Ética em Pesquisa, sob número de parecer: 3.485.858.

## RESULTADOS

Na Figura 1 encontra-se a representação gráfica do modelo de cuidado de enfermagem centrado na família para terapia intensiva neonatal. O modelo é formado por seis conceitos – RN, família, UTIN, saúde-doença, Enfermagem e CCF –, seis pressupostos – 1) A enfermagem deve possibilitar que a UTIN seja acessível, flexível e responsiva às necessidades da família; 2) A enfermagem deve promover o papel central da família no cuidado ao RN, respeitando as opiniões, escolhas e singularidade dos familiares; 3) A construção do vínculo entre enfermeiro e família é essencial para o desenvolvimento do CCF; 4) O enfermeiro precisa conhecer a família para que possa identificar suas necessidades de cuidado, estratégias de enfrentamento e pontos fortes; 5) Para que ocorra comunicação efetiva entre enfermeiro e família, o profissional deve estar aberto, disponível e sem pré-julgamentos durante o diálogo com esta; 6) Deve ser incentivada a parceria entre família e profissionais de saúde para o desenvolvimento do cuidado de forma participativa – e seis fases – ambientando-se, conhecendo-nos, conectando-nos, reconhecendo as necessidades e pontos fortes da família, desenvolvendo o cuidado em parceria com a família e repensando o processo.

No sentido de simplificar o nome do modelo, optou-se por utilizar a sigla AMCORE para representá-lo. Destaca-se que essa sigla foi criada a partir das primeiras sílabas dos nomes das fases da metodologia do cuidado, AM de ambientando-se, CO de conhecendo-nos e conectando-nos e RE de reconhecendo as necessidades e pontos fortes da família. Além de que a sigla AMCORE remete ao que se espera desse modelo: “ancorar”, isto é, estabelecer vínculo e apoiar a família na UTIN.

O diagrama foi inspirado em uma rosa dos ventos, por considerar que o modelo é um guia, uma direção para o cuidado da família em UTIN. O RN e a família estão no centro, pois eles são os receptores do cuidado sustentado pelo modelo, e os demais conceitos, ao seu redor. As fases da metodologia estão nas pontas da rosa, em razão de serem as direções a serem seguidas para o cuidado da família. No Quadro 1 estão apresentadas as definições de cada um dos conceitos que estão representados no diagrama.

**Quadro 1:** Definições dos conceitos que integram o modelo de cuidado de enfermagem centrado na família para terapia intensiva neonatal (AMCORE) Santa Maria, RS, Brasil, 2026.

**RECÉM-NASCIDO** É um ser acolhido pela família, que altera a dinâmica desta, gerando expectativas e preocupações. É um ser frágil, que necessita de cuidados familiares e, por vezes, de profissionais da saúde.
**CUIDADO CENTRADO NA FAMÍLIA** É amplo e complexo, demanda vínculo, respeito pela perspectiva familiar, comunicação efetiva e participação ativa da família e da equipe multiprofissional.
**ENFERMAGEM** Utiliza-se da ciência do cuidado, atuando por meio da assistência, gestão, educação em saúde e pesquisa. Na unidade de terapia intensiva neonatal busca acolher o recém-nascido e sua família, com o objetivo da promoção, manutenção ou recuperação da saúde, e tornar-se, para esta, uma referência de cuidado na equipe multiprofissional.
**UNIDADE DE TERAPIA INTENSIVA NEONATAL** É um ambiente terapêutico, dotado de tecnologia e de uma equipe multiprofissional, a qual presta cuidados ao recém-nascido e sua família. Esse ambiente desperta sentimentos ambíguos como insegurança/segurança e medo/esperança.
**SAÚDE-DOENÇA** É uma experiência subjetiva para a família, já que depende de sua história e condições de vida. É vivenciada pelo recém-nascido e seus familiares, podendo gerar sentimentos e expectativas, que precisam ser considerados para a realização do cuidado.

Nos pressupostos, as pesquisadoras preocuparam-se em salientar a importância que o enfermeiro tem na implementação de cada um deles. Destaca-se que estes foram construídos dedutivamente, tendo por base os oito elementos discutidos na publicação “*Family-centered care for children with special health care needs*”^(11)^ bem como na vivência da pesquisadora como enfermeira de uma unidade de terapia intensiva pediátrica.

As fases da metodologia do modelo de cuidado foram elaboradas pensando em sistematizar como deveria ser o cuidado da família em UTIN. Como embasamento teórico para esta etapa, utilizaram-se outros modelos de cuidado^(8,9,15,16)^, que lançaram luz sobre quais aspectos seriam importantes de serem abordados em cada fase e, sobretudo, o referencial teórico do estudo^(1,11)^, acrescido da experiência prática da pesquisadora. Em todas as fases, aponta-se o momento em que podem ser executadas e quais ações podem ser desenvolvidas para atendê-las. O objetivo não é que sejam estanques, mas sim flexíveis à UTIN em que estão sendo desenvolvidas. Dessa forma, a metodologia apresentada procura oferecer caminhos para o cuidado da família em UTIN. Além disso, na elaboração das fases, as autoras do estudo preocuparam-se em reforçar a importância de a família estar inserida no ambiente da UTIN e nos cuidados com o RN, em razão de que ela ainda precisa construir com este o vínculo, o qual ocorre após o nascimento, e assim desenvolver o seu papel parental, necessário para o cuidado do RN. Na Figura 2, a metodologia do cuidado do modelo é apresentada.

**Figura 2:** Metodologia do cuidado do modelo de cuidado de enfermagem centrado na família para terapia intensiva neonatal (AMCORE).

A a*mbientando-se* foi elencada como primeira fase, por acreditar que a família precisa desse primeiro momento para habituar-se ao ambiente da UTIN e ao RN, além da ambientação necessária por parte do enfermeiro, uma vez que se entende que cada família é única e deve ser cuidada de maneira singular.

Partindo dessa fase, temos a c*onhecendo-nos*, em que o enfermeiro busca conhecer a família e fazer-se conhecer, a fim de iniciar a construção de uma confiança, necessária para o cuidado da família. Na sequência, na fase c*onectando-nos*, são realizadas pelo enfermeiro ações que fomentam a construção do vínculo entre ele e família e entre esta e o RN. A partir das informações obtidas nas fases a*mbientando-se* e c*onhecendo- nos*, o enfermeiro compartilha com a família, por meio de um diálogo aberto, as necessidades identificadas por ele, sendo essa a fase r*econhecendo as necessidades e pontos fortes da família*.

Na fase seguinte, desenvolve-se o cuidado em parceria com a família, definindo, juntamente com ela, estratégias que podem ser desenvolvidas para atender às suas necessidades de cuidado e fortalecer seus pontos fortes. Na fase r*epensando o processo*, o enfermeiro avalia se as estratégias implementadas atenderam às necessidades de cuidado da família, se são necessárias mudanças e/ou adequações e se novas demandas podem ter surgido ao longo deste processo.

Destaca-se que, apesar de as fases estarem descritas em uma ordem, elas não são lineares pois, por vezes, o enfermeiro precisará retornar para uma fase anterior para depois avançar para a seguinte ou, ainda, poderá estar na quinta fase e precisar retornar para a terceira. Entretanto, para que ocorra um CCF, é fundamental que todas as fases sejam observadas, implementadas, repensadas e refletidas.

## DISCUSSÃO

Um modelo de cuidado deve ser constituído pelos conceitos do metaparadigma da Enfermagem e por outros que se fizerem necessários. O metaparadigma é o componente mais abstrato da estrutura do conhecimento de enfermagem. É definido como os conceitos globais que identificam o fenômeno central de interesse da disciplina, as proposições que descrevem os conceitos e as que estabelecem as relações entre eles. Os conceitos que integram o metaparadigma da Enfermagem são ser humano, ambiente, saúde e Enfermagem^(17)^.

Desta forma, foram elaborados os conceitos do metaparadigma, em que o conceito de UTIN refere-se ao ambiente, e os conceitos de RN e família referem-se ao ser humano. Além disso, foi desenvolvido o conceito de CCF considerando a sua relevância para o modelo construído.

Os pressupostos do modelo estão em consonância com os atributos identificados em estudo, que realizou uma análise do conceito de CCF em UTIN. Os atributos são características que tornam os conceitos únicos em relação a outros. Os atributos do conceito de CCF em UTIN identificados são: o cuidado da família – envolve identificar e atender às necessidades de cuidado da família; a participação igualitária da família – a família participa do planejamento e prestação dos cuidados e das tomadas de decisão; a colaboração – os profissionais cooperam com as famílias para a elaboração e implementação dos planos de cuidado; a manutenção do respeito e da dignidade da família – os profissionais reconhecem as diferenças das famílias, incluindo-as na elaboração dos planos de cuidados; e a informação – os profissionais compartilham informações completas às famílias de acordo com as suas especificidades^(18)^.

As fases da metodologia do cuidado foram desenvolvidas para serem um guia para o cuidado da família em UTIN, compreendendo um conjunto de fases interligadas – complementares, simultâneas ou não – que buscam orientar a prática de enfermagem. Na fase a*mbientando-se*, busca-se a ambientação da família à UTIN e ao RN. Quando os pais entram nessa unidade, eles se deparam com um ambiente desconhecido e assustador, rodeado de aparelhos e a imagem de um RN diferente daquele idealizado na gestação, com muitos equipamentos e dispositivos, o que dificulta o contato inicial com ele. Isso implica que os profissionais da equipe apoiem a família, para ajudá-la a superar as dificuldades na consolidação do vínculo^(19)^. Nessa fase, o enfermeiro deve acolher a família, explicar de maneira compreensível sobre a unidade e o RN, esclarecer as suas dúvidas e mostrar-se disponível a ouvi-la.

Outro estudo confirma que, na admissão do RN na UTIN, inicia-se também o acolhimento da família, quando, então, são informados sobre o estado de saúde do RN, orienta-se sobre as rotinas da unidade e são respondidas as suas dúvidas^(20)^. Os pais necessitam saber sobre as rotinas da UTIN, dispositivos médicos e a condição de saúde do filho, o que pode amenizar o medo do desconhecido, sua ansiedade sobre a incerteza da situação, seus sentimentos de exclusão e de impotência^(21)^.

Ainda, o enfermeiro deve intermediar o primeiro contato da família com o RN, oportunizando que o familiar possa tocá-lo e, o mais breve possível, pegá-lo no colo. O contato inicial entre mãe e RN é importante para o desenvolvimento físico, psíquico e intelectual da criança. A internação do RN em UTIN pode comprometer o vínculo frágil criado entre eles. O contato físico, seja por meio do toque, colo ou método canguru, tem sido citado como necessário para os pais desenvolverem vínculo e o seu papel parental^(22,23)^.

Condições para favorecer a presença da família na UTIN devem ser implementadas, tais como: livre acesso dos pais, horários flexíveis para os demais membros da família, acolhimento e comunicação efetiva. Estudo realizado em uma UTIN da Nova Zelândia apontou que não ter uma política de visitação irrestrita para os pais e não ter onde deixar os outros filhos dificulta a presença deles na unidade^(24)^.

Para planejar um cuidado coerente é fundamental conhecer a família. Dessa forma, na fase c*onhecendo-nos*, busca-se conhecer a família, suas expectativas e preocupações em relação à hospitalização do RN na UTIN, suas condições de vida e de saúde, seu apoio social. É preciso ouvi-la com sensibilidade, respeito e disponibilidade, pois essa fase envolve muito mais do que fazer um histórico familiar, trata-se de ouvir o que cada membro da família tem a compartilhar no momento. Outros modelos^(15,25)^ apresentam, entre as suas fases, aquela em que o enfermeiro busca conhecer o ser cuidado, confirmando a importância dessa fase para um modelo.

Ademais, nessa fase, o enfermeiro faz-se conhecer, expondo qual o seu papel e as suas atribuições para a família, explicando como poderá auxiliá-la. Em estudo constatou-se que os pais não sabiam quem eram os enfermeiros e nem as atribuições destes^(26)^. Assim, é preciso que os enfermeiros se apresentem à família, que esta saiba quais são as atribuições desses profissionais e o papel deles dentro da equipe multiprofissional, contribuindo, assim, para a visibilidade da Enfermagem dentro da unidade.

Na fase c*onectando-nos*, busca-se estabelecer vínculo com a família, pois se sabe que isso é fundamental para o desenvolvimento do CCF^(7)^. Esse vínculo pode ser construído por meio da disponibilidade do enfermeiro em ouvir a família, acolhendo suas expectativas e angústias, valorizando o seu conhecimento, valores, crenças e origens culturais, criando condições para que ela participe dos cuidados e do processo de tomada de decisão envolvendo o RN e/ou compartilhando abertamente as informações. Quando os pais se sentem apoiados e confiam nos profissionais de saúde, isso diminui o estresse gerado pela internação em UTIN e aumenta a sua confiança e capacidade em cuidar do seu filho^(27)^.

Na fase r*econhecendo as necessidades e pontos fortes da família*, o enfermeiro estabelece quais são as prioridades de cuidado dos familiares, a partir das informações obtidas nas fases *ambientando-se* e *conhecendo-nos*. Por meio do diálogo, ele compartilha com a família as suas impressões sobre o que acredita ser uma prioridade para ela no momento, ouvindo-a com atenção e respeito, a fim de saber se ela concorda ou não com o que lhe foi exposto, ou ainda, se, na opinião dela existe algo mais importante. Quando se busca um CCF, é preciso ter em mente que as perspectivas e escolhas da família devem sempre ser ouvidas e respeitadas^(1)^. É igualmente importante nessa fase reconhecer quais são as estratégias de enfrentamento e pontos fortes da família, que podem ser incentivados e fortalecidos, a fim de auxiliá-la a superar os desafios decorrentes da hospitalização do RN na UTIN.

Na fase d*esenvolvendo o cuidado em parceria com a família*, busca-se definir com a família as estratégias que serão utilizadas para atender as suas necessidades de cuidado bem como fortalecer seus pontos fortes. A depender da necessidade de cuidado da família, as estratégias podem ser implementadas por ela mesma, pelo enfermeiro conjuntamente com a família, ou ainda, por outros integrantes da equipe multiprofissional.

Os estudos concentram-se na parceria que deve ser desenvolvida com os pais para que eles possam realizar os cuidados do RN, contribuindo, assim, para o desenvolvimento de suas competências, diminuindo o medo e a insegurança de cuidar do filho em casa^(2,28,29)^. Entretanto, a parceria que se almeja na presente fase é aquela que vai além dos cuidados do RN, é a que busca atender às necessidades da família, considerando a sua individualidade e o contexto em que está inserida. Somente assim se atende ao que o CCF se propõe, que é cuidar da família, envolvendo os seus membros no planejamento e na execução dos cuidados^(1)^.

A fase r*epensando o processo* ocorre quando o enfermeiro avalia conjuntamente com a família se as estratégias implementadas na fase anterior atenderam às suas necessidades de cuidado, se são necessárias mudanças e/ou adequações, ou ainda, se novas demandas podem ter surgido. Sabe-se que a internação do RN na UTIN é marcada por incertezas e que a sua condição pode se alterar a qualquer momento, devendo o enfermeiro estar sensível a isso, a fim de auxiliar a família na s mudanças^(19)^.

As limitações do estudo referem-se à coleta dos dados ter sido realizada de maneira remota e em um cenário de estudo no sul do Brasil. Como contribuições para o avanço do conhecimento científico para a área de saúde e enfermagem destaca-se que o modelo de cuidado construído no estudo é uma ferramenta inovadora para a difusão do CCF em UTIN, sendo que a sua metodologia do cuidado oferece elementos que podem guiar os profissionais de enfermagem no cuidado das famílias no ambiente do intensivismo neonatal, de uma maneira simples e adaptável à realidade de cada serviço de saúde. Além disso, o estudo possibilita a visibilidade necessária para o fato de que, para desenvolver um cuidado de qualidade, humanizado e participativo às famílias de RN hospitalizados em UTIN, é necessário que teoria e prática caminhem lado a lado.

## CONCLUSÃO

O AMCORE é constituído pelos conceitos de família, RN, CCF, enfermagem, UTIN e saúde-doença, por pressupostos que têm por base um referencial teórico-prático e uma metodologia do cuidado composta por seis fases, que busca sistematizar o cuidado às famílias nas UTIN. É representado por um diagrama e tem por objetivo não só guiar a prática de enfermeiros de unidades neonatais no que se refere ao cuidado da família, mas também a formação de futuros profissionais, objetivando que os preceitos do CCF sejam cada vez mais difundidos e implementados na prática.

## Data Availability

Todo o conjunto de dados que dá suporte aos resultados deste estudo foi disponibilizado em um Repositório de Dados: http://repositorio.ufsm.br/handle/1/29428.

# Translation: Modelo de atención de enfermería centrado en la familia para terapia intensiva neonatal*

**DOI:** 10.1590/1980-220X-REEUSP-2025-0356es

**Authors:** Jaquiele Jaciára Kegler, Eliane Tatsch Neves, Maria Ribeiro Lacerda

**Affiliations:** 1 Universidade Federal de Santa Maria, Santa Maria, RS, Brasil.; 2 Universidade Federal do Paraná, Curitiba, PR, Brasil.

**Keywords:** Enfermería, Familia, Cuidado Intensivo Neonatal, Modelos de Enfermería, Investigación Cualitativa

## Abstract

**Objetivo:**

Construir un modelo de atención de enfermería centrado en la familia para cuidados intensivos neonatales.

**Método:**

Esta investigación cualitativa, con enfoque participativo, desarrolló todos los componentes de un modelo de atención basado en datos producidos en grupos de discusión a distancia con miembros de un grupo de investigación en el campo de la salud infantil, el marco teórico de la atención centrada en la familia y la experiencia práctica de los investigadores del estudio.

**Resultados:**

Se desarrolló un modelo de atención compuesto por seis conceptos – recién nacido, familia, unidad de cuidados intensivos neonatales, salud-enfermedad, Enfermería y atención centrada en la familia –, seis presupuestos y seis fases – ambientarse, conocerse, conectarse, reconocer las necesidades y fortalezas de la familia, desarrollar el cuidado en conjunto con la familia y repensar el proceso.

**Conclusión:**

Este modelo podría orientar la práctica de los enfermeros en el cuidado de las familias en unidades neonatales y orientar la formación de futuros profesionales.

## INTRODUCCIÓN

El cuidado centrado en la familia (CCF) se refiere a un enfoque para la planificación, prestación y evaluación de la atención sanitaria, que se basa en asociaciones mutuamente beneficiosas entre pacientes, familias y profesionales^(1)^. En pediatría, esto implica que la responsabilidad del cuidado sea compartida por los profesionales de la salud y la familia^(2)^.

Esta atención se basa en cuatro pilares fundamentales que deben aplicarse en cualquier nivel de asistencia y en los contextos neonatal, pediátrico y adulto: dignidad y respeto, intercambio de información, participación y colaboración. Para ello, los profesionales deben escuchar y respetar las decisiones y perspectivas de los pacientes y sus familias (dignidad y respeto); proporcionar información útil, completa e imparcial (intercambio de información), apoyar y animar a los familiares a participar en los cuidados y en la toma de decisiones al nivel que elijan (participación); y contribuir con los pacientes y sus familiares en el desarrollo, la implementación y la evaluación de políticas y programas (colaboración)^(1)^.

Los beneficios del CCF, tanto para los recién nacidos (RN) como para sus padres, están bien documentados en la literatura. Para los bebés, estos beneficios son: mejores resultados en la alimentación y en las puntuaciones de desarrollo neurológico, mayor aumento de peso, disminución del riesgo de retinopatía del prematuro, menor tiempo de hospitalización y menores tasas de reingreso hospitalario; y para los padres: mayor participación, menos ansiedad, depresión y estrés, mayor satisfacción y mejor calidad de vida^(3,4)^. A pesar de ello, el CCF aún es poco comprendido e implementado en las Unidades de Cuidados Intensivos Neonatales (UCIN) de Brasil^(5,6,7)^. Por ello, se decidió desarrollar un modelo de atención que pudiera orientar y fundamentar teóricamente la práctica de los enfermeros en la atención a las familias en las UCIN.

Un modelo de cuidado de enfermería es un conjunto teórico, representado en un diagrama, compuesto por los cuatro conceptos básicos de la enfermería (medio ambiente/sociedad, enfermería, ser humano y salud/enfermedad) y otros que sean necesarios para su comprensión; por supuestos que se basan en un marco teórico-filosófico; y por una metodología de cuidado, cuya función es orientar el cuidado de enfermería mediante la sistematización de las acciones^(8)^.

Los modelos de cuidado son herramientas que pueden utilizarse para guiar la práctica de los enfermeros y, por lo tanto, desmitifican la idea de que la teoría y la práctica no se articulan^(8)^. Además de contribuir al desarrollo de una atención diferenciada y específica, también impulsan el propio conocimiento y desarrollo de la disciplina de Enfermería^(9)^.

De este modo, considerando las lagunas en la formación profesional, al abordar el CCF de forma superficial en las disciplinas de la licenciatura en enfermería, hecho constatado en mi experiencia académica y profesional, la insuficiente educación permanente, las dificultades de los profesionales de enfermería para desarrollar la atención familiar en las unidades neonatales^(5,6,7)^ y la importancia de la CCF para la familia y el recién nacido, se justifica la construcción de un modelo de atención que no solo guíe la práctica de los enfermeros en lo que respecta a la atención familiar en la UCIN, sino que también oriente la formación de futuros profesionales. Además, los conceptos básicos para la aplicación del CCF en la práctica son generales, es decir, no abarcan todas las especificidades de una unidad neonatal y, además, se desarrollaron a partir de la experiencia de investigadores estadounidenses, lo que puede dificultar su aplicación en el contexto brasileño, considerando la diversidad cultural y económica del país.

Otra justificación es la inexistencia de un modelo de esta naturaleza en el contexto brasileño, confirmada a partir de un estudio de revisión integrativa realizado en las bases de datos LILACS, MEDLINE, PubMed y WoS, en noviembre de 2022, con el fin de identificar lo que se ha producido sobre el CCF en unidades neonatales^(10)^. En este sentido, el objetivo de este estudio fue construir un modelo de atención de enfermería centrado en la familia para la terapia intensiva neonatal.

### Referencias Teóricas

La publicación titulada «*Family-centered care for children with special health care needs*»^(11)^ y los conceptos del *Institute for Patient-and Family-centered Care* ^(1)^ sirven de base para las referencias teóricas de este estudio. En la publicación «*Family-centered care for children with special health care needs»*, las autoras presentan ocho elementos considerados clave para la implementación del CCF para familias de niños con algún tipo de necesidad especial, a saber: Reconocer que la familia es la constante en la vida del niño, mientras que los servicios y los profesionales de la salud son transitorios; Facilitar la colaboración entre padres y profesionales en todos los niveles de atención médica; Compartir continuamente información imparcial y completa con los padres sobre el cuidado de sus hijos de manera adecuada y solidaria; Implementar políticas y programas apropiados que sean integrales y brinden apoyo emocional y financiero para atender las necesidades de las familias; Reconocer las fortalezas y la individualidad de la familia y respetar los diferentes métodos de afrontamiento; Comprender e incorporar las necesidades de desarrollo de los bebés, los niños y los adolescentes y sus familias en los sistemas de salud; Fomentar y facilitar el apoyo entre padres; y Garantizar que el *diseño de* los servicios de salud sea flexible, accesible y responda a las necesidades de la familia^(11)^.

Estos elementos constituyeron la base para la construcción de los conceptos de la Atención Centrada en el Paciente y la Familia (CCPF, por sus siglas en inglés): dignidad y respeto, intercambio de información, participación y colaboración, presentados por el Instituto para la Atención Centrada en el Paciente y la Familia. A continuación se describen estos cuatro conceptos:

– Dignidad y respeto: Los profesionales de la salud escuchan y respetan las perspectivas y decisiones de la familia y el paciente. Los conocimientos, valores, creencias y orígenes culturales de la familia y el paciente se incorporan a la planificación y prestación de la atención^(1)^;
– Intercambio de información: los profesionales de la salud comunican y comparten información completa e imparcial con los pacientes y sus familias de manera afirmativa y útil. Los pacientes y sus familias reciben información completa, actualizada y correcta para participar de manera efectiva en la atención y la toma de decisiones^(1)^;
– Participación: se anima y se apoya a los pacientes y a las familias para que participen en la atención y la toma de decisiones al nivel que elijan^(1)^.
– Colaboración: los pacientes, las familias, los profesionales de la salud y los líderes colaboran en el desarrollo de políticas y programas, la implementación y la evaluación; en la investigación; en el diseño del servicio de salud; y en la educación profesional, así como en la promoción de la atención en sí^(1)^.

Otras dos definiciones importantes son la de familia y la del propio CCPF. En la definición presentada por el Instituto para la Atención Centrada en el Paciente y la Familia (2024)^(1)^, los propios pacientes y familiares definen su familia. Y el CCPF se entiende como un enfoque para la planificación, prestación y evaluación de la atención sanitaria, basado en asociaciones mutuamente beneficiosas entre pacientes, familias y profesionales^(1)^.

## MÉTODO

### tipo o Diseño del Estudio

Se trata de una investigación cualitativa, con un enfoque participativo, en la que se elaboraron todos los componentes de un modelo de atención, a saber: los conceptos, los supuestos, la metodología de la atención y un diagrama.

### Población y Criterios de Selección

Para la selección de los participantes, se eligieron como criterios de inclusión: ser enfermero o estudiante de Enfermería y tener al menos dos años de participación en el grupo de investigación Salud del Recién Nacido, Niño, Adolescente y Familia (llamado CRIANDO). Se excluyó a los estudiantes que no hubieran completado el sexto semestre del curso de Enfermería. Este criterio se adoptó teniendo en cuenta que los estudiantes cursan las asignaturas relacionadas con el cuidado del niño y la familia en ese semestre.

Con el fin de identificar a aquellos que cumplían con los criterios de inclusión, se elaboró una tabla con los nombres de todos los miembros del grupo de investigación. A continuación, se contactó con ellos a través de una aplicación de mensajería (WhatsApp), preguntándoles a cada uno de ellos sobre el tiempo de participación en el grupo y, en el caso de los estudiantes, el semestre que estaban cursando en ese momento. Posteriormente, se invitó por correo electrónico a todos los miembros que cumplían los criterios de inclusión.

### Recolección de Datos

Los datos se recopilaron a partir de grupos de discusión remotos realizados con miembros del grupo de investigación CRIANDO. El grupo de discusión es una técnica de recolección de datos mediante la cual el investigador busca la construcción colectiva de ideas^(12)^.

Una vez aceptada la invitación, se envió un correo electrónico tres días antes de la fecha prevista para la realización del grupo de discusión, con información como el objetivo del grupo, el día, la hora y el enlace de acceso, el término de consentimiento libre y esclarecido, así como la actividad que debía realizarse previamente: cada participante debía elegir tres palabras clave para cada concepto (familia, recién nacido, enfermería, UCIN, salud-enfermedad y CCF) que, en su opinión, lo representaran. Las palabras clave elegidas fueron presentadas por cada participante el día programado para cada grupo.

Se organizaron dos grupos distintos, ambos con siete miembros, que se reunieron en diciembre de 2020, en el turno de noche, utilizando la aplicación Google Meet. La discusión del grupo I duró una hora y ocho minutos y la del grupo II, una hora y cuarenta y tres minutos. En ambos grupos participaron todos los niveles académicos: doctorado, maestría y licenciatura.

En los grupos, tras obtener el consentimiento, se presentó a los participantes y se explicó el objetivo del grupo y cómo se organizaría. Posteriormente, cada participante presentó las tres palabras que había elegido para cada concepto, explicando su elección. Al final de la discusión de cada concepto, la investigadora hizo un resumen de las palabras clave de los participantes, que fueron reunidas en un archivo de PowerPoint por la asistente de investigación. Para mantener la organización, se realizó la presentación, discusión y síntesis por concepto, siguiendo el orden RN, UCIN, y así sucesivamente. Los grupos fueron coordinados por la investigadora, autora del proyecto de tesis, y contaron con la participación de una asistente de investigación, previamente capacitada, que era estudiante de licenciatura en Enfermería y también becaria del proyecto.

### Análisis y Tratamiento de los Datos

Las grabaciones de audio de los grupos de discusión se transcribieron en un documento de Word y se analizaron, tomando como referencia el análisis temático inductivo^(13)^, siguiendo los siguientes pasos:

– Familiarización con los datos: se realizaron lecturas y relecturas exhaustivas del material transcrito y se destacaron las palabras clave de cada concepto en el texto. Para ello, se utilizaron seis colores diferentes;
– Generación de códigos iniciales: todas las palabras clave resaltadas en el texto se agruparon en un cuadro, se leyeron exhaustivamente y se clasificaron por colores según su afinidad temática;
– Generación de temas: las palabras clave resaltadas con el mismo color se agruparon en temas;
– Revisión de los temas: se revisaron los temas para confirmar si las palabras clave agrupadas en cada tema lo representaban adecuadamente;
– Generación de conceptos: se agruparon los temas de cada concepto y, con la ayuda de los conectores del idioma portugués, así como basándose en el marco teórico de la tesis, se construyeron los conceptos.

El proceso de análisis que sustentó la elaboración de cada uno de los conceptos se detalla en un artículo publicado en la Revista Brasileira de Enfermagem^(14)^.

En un segundo momento, se realizaron dos grupos de discusión más para validar los conceptos construidos y/o sugerir modificaciones. Los grupos se llevaron a cabo en junio de 2021, a través de Google Meet, en los que participaron 11 de los 14 miembros de los grupos anteriores. En ese momento, 3 de ellos no pudieron participar por motivos personales. El grupo I (cinco miembros) tuvo una duración de una hora y quince minutos y el grupo II (seis miembros), de una hora y once minutos. En estos grupos, los participantes presentaron sus sugerencias al resto y analizaron qué aspectos de los conceptos necesitaban ajustes. Cabe destacar que los conceptos se enviaron previamente por correo electrónico para su análisis. En todos ellos se sugirieron modificaciones, que fueron analizadas y discutidas por el equipo de investigación, formado por la investigadora doctoral, la tutora y la co-tutora, quienes reformularon nuevamente los conceptos.

Los supuestos y la metodología del cuidado se elaboraron basándose en el marco teórico de la tesis^(1,11)^ y la experiencia práctica de las investigadoras. A lo largo de las reuniones del equipo de investigación, los componentes del modelo de cuidado se fueron reformulando para que reflejaran el marco teórico y fueran claros y coherentes entre sí y con la práctica.

Tras su elaboración, los conceptos, supuestos y metodología de la atención se enviaron para su revisión gramatical, con el fin de analizar la cohesión y la coherencia textual. A continuación, el equipo de investigación los reestructuró a partir de las sugerencias de la revisora.

También se elaboró un diagrama como elemento de un modelo de atención de enfermería. De esta manera, durante el proceso de elaboración de los conceptos, la investigadora había dibujado en una hoja A4 un diagrama preliminar de las primeras ideas sobre cómo debían relacionarse los conceptos. Posteriormente, el día de los grupos de discusión, en el que se validaron los conceptos, los participantes también presentaron y explicaron el diagrama que habían dibujado para representar los conceptos. Cabe destacar que en el mismo correo electrónico en el que se envió el archivo *Word* con los conceptos, se solicitó, como tarea a realizar previamente al día del grupo, que los participantes dibujaran en una hoja A4 cómo representarían los conceptos en un diagrama, enviándolo a través de la aplicación de mensajería (WhatsApp).

Estos diagramas sirvieron de inspiración, junto con las ideas discutidas por el equipo de investigación, para la creación de la ilustración (Figura 1), que representa el modelo de atención, realizada por un diseñador gráfico profesional contratado por la investigadora doctoral.

**Figura 1:** Representación gráfica del modelo de cuidado de enfermería centrado en la familia para terapia intensiva neonatal (AMCORE).

Una vez elaborado el modelo de atención, se realizaron algunas reformulaciones para que fuera fácil de entender, sencillo y atractivo para los enfermeros. Para ello, se utilizaron colores e ilustraciones para representar las fases de la metodología, también creadas por el diseñador gráfico profesional, así como se realizaron algunas supresiones y se añadieron las definiciones de los componentes de un modelo de atención. El objetivo era crear un material de interés para los enfermeros.

### Aspectos Éticos

El estudio se desarrolló siguiendo la Resolución n.º 466/2012 y fue aprobado por el Comité de Ética en Investigación, con el número de dictamen: 3.485.858.

## RESULTADOS

En la Figura 1 se encuentra la representación gráfica del modelo de atención de enfermería centrado en la familia para terapia intensiva neonatal. El modelo se compone de seis conceptos (recién nacido, familia, UCIN, salud-enfermedad, enfermería y CCF) y seis supuestos: 1) La enfermería debe permitir que la UCIN sea accesible, flexible y receptiva a las necesidades de la familia; 2) La enfermería debe promover el papel central de la familia en el cuidado del recién nacido, respetando las opiniones, las elecciones y la singularidad de los familiares; 3) La construcción del vínculo entre el enfermero y la familia es esencial para el desarrollo del CCF; 4) El enfermero necesita conocer a la familia para poder identificar sus necesidades de cuidado, estrategias de afrontamiento y puntos fuertes; 5) Para que se produzca una comunicación eficaz entre el enfermero y la familia, el profesional debe estar abierto, disponible y sin prejuicios durante el diálogo con esta; 6) Se debe fomentar la colaboración entre la familia y los profesionales de la salud para el desarrollo de la atención de forma participativa, en seis fases: ambientándonos, conociéndonos, conectándonos, reconociendo las necesidades y puntos fuertes de la familia, desarrollando la atención en colaboración con la familia y replanteándonos el proceso.

Con el fin de simplificar el nombre del modelo, se optó por utilizar el acrónimo AMCORE para representarlo. Cabe destacar que este acrónimo se creó a partir de las primeras sílabas de los nombres de las fases de la metodología de cuidados: AM de ambientándonos, CO de conociéndonos y conectándonos y RE de reconociendo las necesidades y fortalezas de la familia. Además, la sigla AMCORE remite a lo que se espera de este modelo: «anclar», es decir, establecer vínculos y apoyar a la familia en la UCIN.

El diagrama se inspiró en una rosa de los vientos, ya que se considera que el modelo es una guía, una orientación para el cuidado de la familia en la UCIN. El recién nacido y la familia se encuentran en el centro, ya que son los receptores del cuidado que sustenta el modelo, y los demás conceptos se sitúan a su alrededor. Las fases de la metodología se encuentran en los extremos de la rosa, ya que son las direcciones que se deben seguir para el cuidado de la familia. En el Cuadro 1 se presentan las definiciones de cada uno de los conceptos que se representan en el diagrama.

**Cuadro 1:** Definiciones de los conceptos que integran el modelo de atención de enfermería centrada en la familia para terapia intensiva neonatal (AMCORE) – Santa Maria, RS, Brasil, 2026.

**RECÉN NACIDO** Es un ser acogido por la familia, lo que altera la dinámica de esta, generando expectativas y preocupaciones. Es un ser frágil, que necesita cuidados familiares y, en ocasiones, de profesionales de la salud.
**CUIDADO CENTRADO EN LA FAMILIA** Es amplio y complejo, exige vínculos, respeto por la perspectiva familiar, comunicación efectiva y participación activa de la familia y del equipo multidisciplinario.
**ENFERMERÍA** Utiliza la ciencia del cuidado, actuando a través de la asistencia, la gestión, la educación en salud y la investigación. En la unidad de terapia intensiva neonatal se busca acoger al recién nacido y a su familia, con el objetivo de promover, mantener o recuperar la salud, y convertirse, para ello, en un referente de cuidado en el equipo multidisciplinario.
**UNIDAD DE TERAPIA INTENSIVA NEONATAL** Es un entorno terapéutico, dotado de tecnología y un equipo multidisciplinario, que brinda cuidados al recién nacido y a su familia. Este entorno despierta sentimientos ambiguos como inseguridad/seguridad y miedo/esperanza.
**SALUD-ENFERMEDAD** Es una experiencia subjetiva para la familia, ya que depende de su historia y condiciones de vida. Es vivida por el recién nacido y sus familiares, y puede generar sentimientos y expectativas que deben tenerse en cuenta para la realización de los cuidados.

En los supuestos, las investigadoras se preocuparon por destacar la importancia que tiene el enfermero en la implementación de cada uno de ellos. Cabe destacar que estos se construyeron de forma deductiva, basándose en los ocho elementos discutidos en la publicación «*Family-centered care for children with special health care needs*»^(11)^, así como en la experiencia de la investigadora como enfermera de una unidad de terapia intensiva pediátrica.

Las fases de la metodología del modelo de atención se elaboraron con el fin de sistematizar cómo debería ser la atención a la familia en la UCIN. Como base teórica para esta etapa, se utilizaron otros modelos de atención^(8,9,15,16)^, que arrojaron luz sobre los aspectos que sería importante abordar en cada fase y, sobre todo, el marco teórico del estudio^(1,11)^, al que se sumó la experiencia práctica de la investigadora. En todas las fases se señala el momento en que pueden ejecutarse y qué acciones pueden desarrollarse para atenderlas. El objetivo no es que sean rígidas, sino flexibles a la UCIN en la que se están desarrollando. De este modo, la metodología presentada busca ofrecer vías para el cuidado de la familia en la UCIN. Además, en la elaboración de las fases, las autoras del estudio se preocuparon por reforzar la importancia de que la familia se integre en el entorno de la UCIN y en el cuidado del recién nacido, ya que aún necesita construir con él el vínculo, que se produce después del nacimiento, y así desarrollar su papel parental, necesario para el cuidado del recién nacido. En la Figura 2 se presenta la metodología de cuidado del modelo.

**Figura 2:** Metodología del cuidado del modelo de cuidado de enfermería centrado en la familia para terapia intensiva neonatal (AMCORE).

La *adaptación al entorno* se consideró la primera fase, ya que se cree que la familia necesita este primer momento para acostumbrarse al entorno de la UCIN y al recién nacido, además de la adaptación necesaria por parte del enfermero, ya que se entiende que cada familia es única y debe ser atendida de manera singular.

A partir de esta fase, tenemos la fase de *conocernos*, en la que el enfermero busca conocer a la familia y darse a conocer, con el fin de comenzar a construir la confianza necesaria para el cuidado de la familia. A continuación, en la fase de*conectarnos*, el enfermero lleva a cabo acciones que fomentan la construcción del vínculo entre él y la familia y entre esta y el recién nacido. A partir de la información obtenida en las fases de*acostumbrarnos* y *conocernos*, el enfermero comparte con la familia, a través de un diálogo abierto, las necesidades identificadas por él, siendo esta la fase de*reconocer las necesidades y puntos fuertes de la familia*.

En la siguiente fase, se desarrolla el cuidado en colaboración con la familia, definiendo junto con ella estrategias que pueden desarrollarse para atender sus necesidades de cuidado y fortalecer sus puntos fuertes. En la fase de replanteamiento del proceso, el enfermero evalúa si las estrategias implementadas han atendido las necesidades de cuidado de la familia, si son necesarios cambios y/o ajustes y si pueden haber surgido nuevas demandas a lo largo de este proceso.

Cabe destacar que, aunque las fases se describen en un orden, no son lineales, ya que, en ocasiones, el enfermero tendrá que volver a una fase anterior para luego avanzar a la siguiente o, incluso, puede estar en la quinta fase y tener que volver a la tercera. Sin embargo, para que se produzca un CCF, es fundamental que todas las fases se observen, implementen, repensen y reflexionen.

## DISCUSIÓN

Un modelo de atención debe estar constituido por los con ceptos del metaparadigma de la Enfermería y otros que sean necesarios. El metaparadigma es el componente más abstracto de la estructura del conocimiento de la enfermería. Se define como los conceptos globales que identifican el fenómeno central de interés de la disciplina, las proposiciones que describen los conceptos y las que establecen las relaciones entre ellos. Los conceptos que integran el metaparadigma de la enfermería son ser humano, entorno, salud y enfermería^(17)^.

De esta forma, se elaboraron los conceptos del metaparadigma, en los que el concepto de UCIN se refiere al entorno, y los conceptos de recién nacido y familia se refieren al ser humano. Además, se desarrolló el concepto de CCF considerando su relevancia para el modelo construido.

Los supuestos del modelo están en consonancia con los atributos identificados en el estudio, que realizó un análisis del concepto de CCF en la UCIN. Los atributos son características que hacen que los conceptos sean únicos en relación con otros. Los atributos del concepto de CCF en la UCIN identificados son: el cuidado de la familia, que implica identificar y atender las necesidades de cuidado de la familia; la participación igualitaria de la familia, que participa en la planificación y prestación de cuidados y en la toma de decisiones; la colaboración, en la que los profesionales cooperan con las familias para elaborar e implementar los planes de cuidado; el mantenimiento del respeto y la dignidad de la familia: los profesionales reconocen las diferencias entre las familias y las incluyen en la elaboración de los planes de cuidados; y la información: los profesionales comparten información completa con las familias de acuerdo con sus especificidades^(18)^.

Las fases de la metodología de cuidados se desarrollaron para servir de guía para el cuidado de la familia en la UCIN, y comprenden un conjunto de fases interrelacionadas —complementarias, simultáneas o no— que buscan orientar la práctica de la enfermería. En la fase de *adaptación al entorno*, se busca que la familia se adapte a la UCIN y al recién nacido. Cuando los padres entran en esta unidad, se encuentran con un entorno desconocido y aterrador, rodeado de aparatos y con la imagen de un recién nacido diferente al idealizado durante el embarazo, con muchos equipos y dispositivos, lo que dificulta el contacto inicial con él. Esto implica que los profesionales del equipo apoyen a la familia para ayudarla a superar las dificultades en la consolidación del vínculo^(19)^. En esta fase, el enfermero debe acoger a la familia, explicar de manera comprensible sobre la unidad y el recién nacido, aclarar sus dudas y mostrarse dispuesto a escucharla.

Otro estudio confirma que, al ingresar al recién nacido en la UCIN, también comienza la acogida de la familia, cuando se les informa sobre el estado de salud del recién nacido, se les orienta sobre las rutinas de la unidad y se responden sus dudas^(20)^. Los padres necesitan conocer las rutinas de la UCIN, los dispositivos médicos y el estado de salud de su hijo, lo que puede aliviar el miedo a lo desconocido, su ansiedad por la incertidumbre de la situación, sus sentimientos de exclusión e impotencia^(21)^.

Además, el enfermero debe intermediar en el primer contacto de la familia con el recién nacido, dando la oportunidad a los familiares de tocarlo y, lo antes posible, cogerlo en brazos. El contacto inicial entre la madre y el recién nacido es importante para el desarrollo físico, psíquico e intelectual del niño. La hospitalización del recién nacido en la UCIN puede comprometer el frágil vínculo creado entre ellos. El contacto físico, ya sea mediante el tacto, el abrazo o el método canguro, se ha citado como necesario para que los padres desarrollen el vínculo y su papel parental^(22,23)^.

Deben implementarse condiciones que favorezcan la presencia de la familia en la UCIN, tales como: libre acceso de los padres, horarios flexibles para los demás miembros de la familia, acogida y comunicación efectiva. Un estudio realizado en una UCIN de Nueva Zelanda señaló que no tener una política de visitas sin restricciones para los padres y no tener dónde dejar a los otros hijos dificulta su presencia en la unidad^(24)^.

Para planificar una atención coherente, es fundamental conocer a la familia. De este modo, en la fase de «conocernos», se busca conocer a la familia, sus expectativas y preocupaciones en relación con la hospitalización del recién nacido en la UCIN, sus condiciones de vida y salud, y su apoyo social. Es necesario escucharla con sensibilidad, respeto y disponibilidad, ya que esta fase implica mucho más que hacer un historial familiar, se trata de escuchar lo que cada miembro de la familia tiene para compartir en ese momento. Otros modelos^(15,25)^ presentan, entre sus fases, aquella en la que el enfermero busca conocer al paciente, confirmando la importancia de esta fase para un modelo.

Además, en esta fase, el enfermero se da a conocer, exponiendo su papel y sus funciones a la familia, explicando cómo puede ayudarla. En un estudio se constató que los padres no sabían quiénes eran los enfermeros ni cuáles eran sus funciones^(26)^. Por lo tanto, es necesario que los enfermeros se presenten a la familia, que esta conozca cuáles son las atribuciones de estos profesionales y su papel dentro del equipo multidisciplinario, contribuyendo así a la visibilidad de la enfermería dentro de la unidad.

En la fase «conectándonos», se busca establecer un vínculo con la familia, ya que se sabe que esto es fundamental para el desarrollo del CCF^(7)^. Este vínculo puede construirse mediante la disponibilidad del enfermero para escuchar a la familia, acogiendo sus expectativas y angustias, valorando sus conocimientos, valores, creencias y orígenes culturales, creando las condiciones para que participe en los cuidados y en el proceso de toma de decisiones que involucran al recién nacido y/o compartiendo abiertamente la información. Cuando los padres se sienten apoyados y confían en los profesionales de la salud, esto disminuye el estrés generado por la hospitalización en la UCIN y aumenta su confianza y capacidad para cuidar a su hijo^(27)^.

En la fase de *reconocer las necesidades y fortalezas de la familia*, el enfermero establece cuáles son las prioridades de cuidado de los familiares, a partir de la información obtenida en las fases de *adaptarse* y *conocernos*. A través del diálogo, comparte con la familia sus impresiones sobre lo que cree que es una prioridad para ella en ese momento, escuchándola con atención y respeto, con el fin de saber si está de acuerdo o no con lo que se le ha expuesto, o si, en su opinión, hay algo más importante.

Cuando se busca un CCF, hay que tener en cuenta que las perspectivas y elecciones de la familia siempre deben ser escuchadas y respetadas^(1)^. En esta fase, es igualmente importante reconocer cuáles son las estrategias de afrontamiento y los puntos fuertes de la familia, que pueden ser incentivados y fortalecidos, con el fin de ayudarla a superar los retos derivados de la hospitalización del recién nacido en la UCIN.

En la fase de *desarrollo del cuidado en colaboración con la familia*, se busca definir con la familia las estrategias que se utilizarán para atender sus necesidades de cuidado, así como fortalecer sus puntos fuertes. Dependiendo de las necesidades de cuidado de la familia, las estrategias pueden ser implementadas por ella misma, por el enfermero junto con la familia o por otros miembros del equipo multidisciplinario.

Los estudios se centran en la colaboración que debe desarrollarse con los padres para que puedan cuidar al recién nacido, contribuyendo así al desarrollo de sus competencias y disminuyendo el miedo y la inseguridad de cuidar a su hijo en casa^(2,28,29)^. Sin embargo, la colaboración que se busca en la presente fase va más allá del cuidado del recién nacido, ya que pretende atender las necesidades de la familia, teniendo en cuenta su individualidad y el contexto en el que se encuentra. Solo así se cumple lo que se propone el CCF, que es cuidar a la familia, involucrando a sus miembros en la planificación y la ejecución de los cuidados^(1)^.

La fase de «replanteamiento del proceso» se produce cuando el enfermero evalúa conjuntamente con la familia si las estrategias implementadas en la fase anterior han satisfecho sus necesidades de cuidados, si son necesarios cambios y/o ajustes, o si han surgido nuevas demandas. Se sabe que la hospitalización del recién nacido en la UCIN está marcada por incertidumbres y que su estado puede cambiar en cualquier momento, por lo que el enfermero debe ser sensible a ello para ayudar a la familia en los cambios^(19)^.

Las limitaciones del estudio se refieren a que la recolección de datos se realizó de forma remota y en un escenario de estudio en el sur de Brasil. Como contribución al avance del conocimiento científico en el área de la salud y la enfermería, cabe destacar que el modelo de atención construido en el estudio es una herramienta innovadora para la difusión de la CCF en la UCIN, ya que su metodología de atención ofrece elementos que pueden guiar a los profesionales de enfermería en la atención a las familias en el entorno de la terapia intensiva neonatal, de una manera sencilla y adaptable a la realidad de cada servicio de salud. Además, el estudio permite visibilizar el hecho de que, para desarrollar una atención de calidad, humanizada y participativa a las familias de los recién nacidos hospitalizados en la UCIN, es necesario que la teoría y la práctica vayan de la mano.

## CONCLUSIÓN

El AMCORE se basa en los conceptos de familia, recién nacido, CCF, enfermería, UCIN y salud-enfermedad, en supuestos que se basan en un marco teórico-práctico y una metodología de cuidados compuesta por seis fases, que busca sistematizar los cuidados a las familias en las UCIN.

Se representa mediante un diagrama y tiene como objetivo no solo guiar la práctica de los enfermeros de las unidades neonatales en lo que se refiere al cuidado de la familia, sino también la formación de futuros profesionales, con el fin de que los preceptos del CCF se difundan y se implementen cada vez más en la práctica.

## Data Availability

Todo el conjunto de datos que respalda los resultados de este estudio se ha puesto a disposición en un repositorio de datos: http://repositorio.ufsm.br/handle/1/29428.
